# Effects of Intermittent Neck Cooling During Repeated Bouts of High-Intensity Exercise

**DOI:** 10.3390/sports4030038

**Published:** 2016-06-29

**Authors:** Andrew J. Galpin, James R. Bagley, Blake Whitcomb, Leonard D. Wiersma, Jakob Rosengarten, Jared W. Coburn, Daniel A. Judelson

**Affiliations:** 1 Center for Sport Performance, Department of Kinesiology, California State University, Fullerton, CA 92834, USA; blake.whitcomb@gmail.com (B.W.); lwiersma@fullerton.edu (L.D.W.); jakoblpc@googlemail.com (J.R.); jcoburn@fullerton.edu (J.W.C.); dan.judelson@nike.com (D.A.J.); 2 Department of Kinesiology, San Francisco State University, San Francisco, CA 94132, USA; jrbagley@sfsu.edu

**Keywords:** mixed martial arts, recovery, combat sports, endurance, performance, ice

## Abstract

The purpose of this investigation was to determine the influence of intermittent neck cooling during exercise bouts designed to mimic combat sport competitions. Participants (*n* = 13, age = 25.3 ± 5.0 year height = 176.9 ± 7.5 cm, mass = 79.3 ± 9.0 kg, body fat = 11.8% ± 3.1%) performed three trials on a cycle ergometer. Each trial consisted of two, 5-min high-intensity exercise (HEX) intervals (HEX1 and HEX2—20 s at 50% peak power, followed by 15 s of rest), and a time to exhaustion (TTE) test. One-minute rest intervals were given between each round (RI1 and RI2), during which researchers treated the participant’s posterior neck with either (1) wet-ice (ICE); (2) menthol spray (SPRAY); or (3) no treatment (CON). Neck (T_NECK_) and chest (T_CHEST_) skin temperatures were significantly lower following RI1 with ICE (vs. SPRAY). Thermal sensation decreased with ICE compared to CON following RI1, RI2, TTE, and a 2-min recovery. Rating of perceived exertion was also lower with ICE following HEX2 (vs. CON) and after RI2 (vs. SPRAY). Treatment did not influence TTE (68.9 ± 18.9s). The ability of intermittent ICE to attenuate neck and chest skin temperature rises during the initial HEX stages likely explains why participants felt cooler and less exerted during equivalent HEX bouts. These data suggest intermittent ICE improves perceptual stress during short, repeated bouts of vigorous exercise.

## 1. Introduction

The scientific community has recently drawn interest in the complex physiological demands of combat sports (e.g., wrestling, boxing, judo) [1]. Their energetic requirements and activity patterns differ from steady-state sports such as cycling or running whereby they require alternating bouts of maximal exertion (usually 5–15 s) and active rest (usually 5–40 s), repeated for 2–6 min, per round, with most competitions requiring 3–10 rounds [1,2,3,4]. Causes of fatigue during these activities, or similar forms of high-intensity exercise (HEX), are multifaceted as various physiological, and perceptual inputs interact to control both physical capacity and the desire to perform it [5,6,7]. Specifically, a combination of body temperature and the conscious perception of thermal distress [8], thermal sensation (TS), and the rating of perceived exertion (RPE) [9] combine to determine exercise workload/performance [5,6,7,10]. 

Reducing skin temperature in thermally sensitive areas (face, hands, specific exercising muscles, or a combination of these) may reduce TS, thermal discomfort, and RPE, which improves exercise performance and perception [7,10,11]. Applying cooling agents (e.g., ice packs) to these locations before or during the rest intervals (RI) of HEX (i.e., “intermittent cooling”) has been shown to increase exercise power output [12] and time to fatigue [10,13,14]. Unfortunately, cooling these areas is not practical for combat sports due to the number of muscles involved, the equipment, and sport rules [9]. Coaches typically circumvent this problem by placing an ice bag on the back of an athlete’s necks between rounds. Neck cooling typically enhances performance during low- [15,16,17] and moderate-intensity exercise (≤70% VO_2_max) [16,17]. However, little is known about its effectiveness during HEX.

A growing collection of parallel research strongly supports thermal perception as a thermoregulatory controller [7,8,18]. Thus, researchers are now also investigating the effects of perceived (i.e., non-thermal) cooling on thermoregulation and performance [5,6,7,18], particularly when applied to the neck. The early indications suggest that non-thermal cooling (usually menthol sprays) does not alter core and skin temperatures, skin blood flow, or skin sweat rate [5], but does promote positive changes in TS and RPE.

Taken together, while the literature supports a relationship between body cooling and exercise performance, it is still unclear (1) if intermittent neck cooling during HEX alters performance, TS, or the perception of difficulty, and (2) if these alterations are a result of actual physical cooling, or simply the perception of cooling. Therefore, the purpose of this study was to investigate physiological and perceptual effects of intermittent neck cooling during the rest periods of repeated HEX bouts designed to mimic combat sport competition energetic demands.

## 2. Materials and Method

### 2.1. Study Design

**Figure 1:**
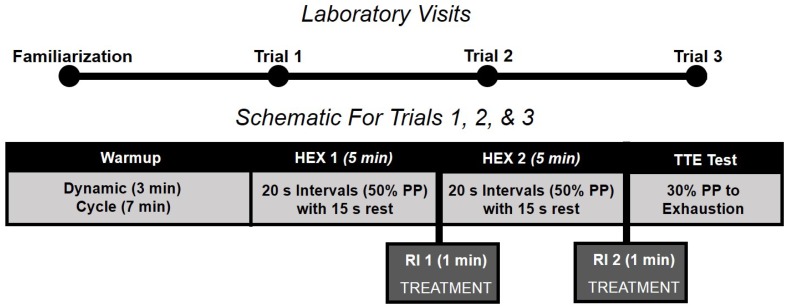
Laboratory visits timeline and schematic showing repeated high-intensity exercise bouts. For each visit, the TREATMENT included either ICE (wet ice), SPRAY (menthol spray), or CON (control, no treatment). Abbreviations: HEX: high-intensity bout of exercise; PP: peak power; RI: rest interval; TTE: time to exhaustion.

Participants reported to the laboratory on four separate occasions (each separated by ≥48 and ≤168 h), which included one familiarization and three experimental trials (Figure 1). Each trial consisted of repeated bouts of HEX cycling. During the rest intervals (RI) between acute HEX bouts, participants had (A) a wet-ice bag (ICE) [19], (B) a menthol spray (SPRAY), or (C) nothing (CON) placed on their cervical spine. This was designed to mimic a typical 3-round combat sport event (i.e., 3 × 5-min rounds, with 1-min rest between rounds). Only one treatment (ICE, SPRAY, or CON) was used per experimental trial, and the order was randomized.

### 2.2. Experimental Controls

All trials took place in the Exercise Physiology Laboratory (maintained at 25.0 ± 1.0 °C, 53.0% ± 1.0% relative humidity) with the same technicians, at the same time of the day (within participant). Participants were asked to not exercise the 24 h before each trial, but were encouraged to maintain their normal training routine throughout the duration of the study. Participants were instructed to consume a diet similar to their first trial for all subsequent trials. To verify consistency, hydration and food logs were recorded 24 h prior to each trial. Participants were further instructed to consume 500 mL of water the night before and 500 mL in the morning before the start of the trial. Height, mass, and body fat percentage (via bioelectrical impedance analysis) was recorded prior to each trial. To minimize confounding influences of motivation, participants were given no feedback on their performance, nor did they receive verbal (or any other) encouragement at any point of the study.

### 2.3. Participants

Physically active males (*n* = 13, age = 25.3 ± 5.0 year, height = 176.9 ± 7.5 cm, mass = 79.3 ± 9.0 kg, body fat = 11.8% ± 3.1%) without previous experience with neck cooling were recruited for this investigation. In order to participate, volunteers were required to have performed repeated HEX bouts (which we defined as repeated bouts of ≥90% maximal perceived exertion and/or heart rate) ≥3 times per week for at least six weeks prior to testing, and to have been free of any current joint, musculoskeletal, or neuromuscular injuries. Prior to participation, volunteers signed an informed consent form and filled out a physical activity questionnaire previously approved by the California State University, Fullerton Institutional Review Board.

### 2.4. Familiarization and Experimental Trials

Before every trial (familiarization and three experimental), participants performed a 10-min warm-up consisting of 3 min (total) of knee hugs, toe touches, lunges, power skips, and side shuffles, and 7 min of cycling at 50 Watts (self-selected cadence). For the familiarization trial, participants followed the warm-up with a 10-s Wingate test (to measure peak power (PP)), rested for 10 min, and then completed the full experimental trial (including scale reading practice and wearing of the metabolic cart headgear or mouthpiece). For the experimental trials, 1 min of rest was given between the end of the warm-up and beginning of the HEX.

Each experimental trial was performed on a cycle ergometer (Ergomedic 839E, Monark Exercise AB, Sweden) and consisted of two, 5-min HEX bouts (20 s maximal cadence cycling, plus 15 s passive rest) at 50% PP, plus a single bout to exhaustion (TTE) at 30% PP. Pilot work suggested 50% PP allowed participants to reach maximal effort within a 20-s interval, while still being physically capable of completing the full protocol. The work-to-rest ratio of 20:15 s was chosen because it is a standard protocol used among HEX practitioners, and it also represents the typical activity pattern observed during combat sport competitions [3]. Exhaustion during the TTE was defined as the participant’s inability to pedal ≥60 revolutions per min for more than 5 consecutive seconds. Pilot data also suggested 30% PP would allow most participants to reach exhaustion between 1–5 min, which is the length of most combat sport rounds.

A 1-min passive RI separated all three bouts of exercise (the two HEX and the one TTE). During the RI, participants remained on the bike but were not allowed to cycle, as combat sport athletes typically sit passively on a stool during competition RI. However, during the RI between HEX round 1 (HEX1) and HEX round 2 (HEX2), and between HEX2 and TTE, the posterior neck was treated with either ICE, SPRAY, or CON. The treatment (ICE, SPRAY, or CON) was consistent within each trial, but the order of each trial was randomized (e.g., Participant 1 received SPRAY Day 1; CON, Day 2; ICE, Day 3, while Participant 2 received CON, Day 1; ICE, Day 2; SPRAY, Day 3).

For ICE, participants had a researcher hold an ice bag (Mueller Sport Medicine, Inc., Prairie Du Sac, Wisconsin) filled with 1 quart of ice (immediately from freezer) and 600 mL of room temperature water on the back of the neck (in direct contact with only the skin) [19]. This method mimics the cooling technique frequently used in combat sport competitions. SPRAY was achieved by having a researcher apply 2–4 mL of a commercially available 8% menthol spray (Stopain: DRJ Group, Inc., Hazelton, Pennsylvania) directly on the participant’s neck at the beginning of RI1 and RI2. The spray was wiped off the neck with a towel immediately following RI1 and RI2 to prevent potential residual influences on subsequent exercise bouts (i.e., HEX2 and TTE) [7]. Participants were not informed of the reason for including the SPRAY treatment. Participants received no treatment during either RI1 or RI2 during the CON trial.

### 2.5. Skin Temperature

Skin temperature was recorded every second from the beginning of the cycling warm-up until the end of recovery. Four calibrated surface thermistors (DS1922L-F5# Thermochron, Ibutton, Whitewater, Wisconsin) were secured in place with surgical tape to the neck (T_NECK_), the right side of the pectoralis major (T_CHEST_), the upper arm (T_ARM_), and the quadriceps (T_LEG_). Data were reported at the same time points as TS and RPE. Core temperature was not reported, as similar cooling research indicated that it would not be influenced by our treatments [7,17,18].

### 2.6. Expired Gas and Heart Rate 

Expired gases were collected with a metabolic cart (Parvo Medics 2400, Sandy, Utah) throughout every trial (i.e., cycling warm-up through recovery). A chest monitor (Polar FS1, Lake Success, New York) was used to record heart rate (HR) through the same period. Expired gas and HR data were recorded every 15 s from the beginning of the cycling warm-up until the end of recovery.

### 2.7. Perceptual Measures

TS and rating of RPE were measured by having participants point to visual scales (1) immediately (<2 s) following HEX1/start of RI1, (2) immediately following RI1/the start of HEX2, (3) immediately following HEX2/start of RI2, (4) immediately following RI2/the start of TTE, (5) immediately following TTE, and (6) 2 min after the end of TTE (recovery). TS was rated with a 9-point scale, ranging from 0 (unbearably cold) to 8 (unbearably hot), with 4 as comfortable (neutral). RPE was measured on the 15-grade Borg scale ranging from 6 (no exertion) to 20 (maximal exertion).

### 2.8. Statistical Analysis

All statistical analyses were performed on IBM SPSS Statistics 20.0. Alpha was set at *p* < 0.05. TTE was analyzed using a 3 × 1 (treatment x time) repeated measures ANOVA. A 3 × 6 (treatment x time) factorial ANOVA was used to analyze TS, RPE, skin temperature, VO_2_, RER, VE, and HR. Where appropriate, LSD post hoc test comparisons were used. Standardized effect size (Cohen’s d; *d*) analyses were used in interpreting the magnitude of differences between treatments. An effect size was classified as trivial (<0.20), small (0.20–0.49), moderate (0.50–0.79), or large (>0.80) as expressed by dividing the mean difference by the between-participant SD. All data are presented as mean ± SD.

## 3. Results

### 3.1. Skin Temperature

**Figure 2:**
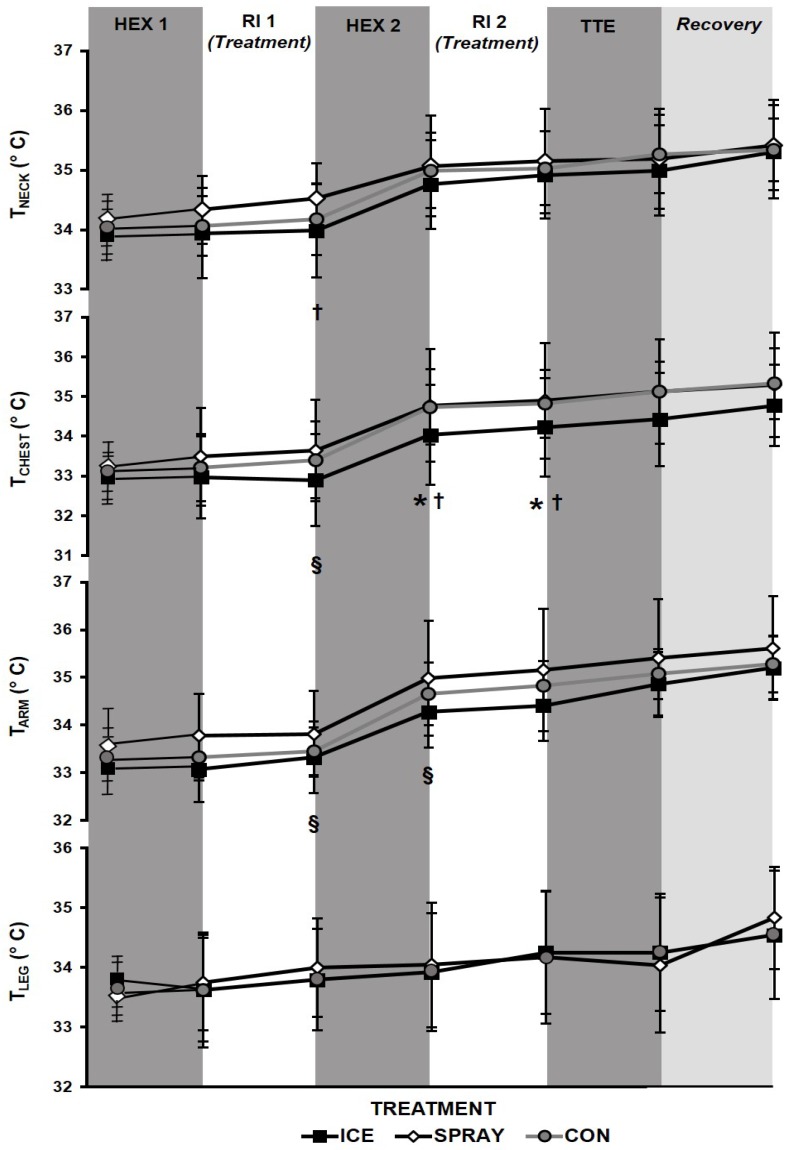
Skin temperatures (T_NECK_, neck; T_CHEST_, chest) during exercise bouts (HEX: high-intensity exercise interval; TTE: time-to-exhaustion) with intermittent rest intervals (RI) and neck cooling treatments (ICE: “wet ice” bag; SPRAY: non-thermal cool spray; or CON: control). Significance among treatments: * ICE vs. CON (*p* < 0.05), ^§^ ICE vs. CON (*trend*; *p* ≤ 0.08), ^†^ ICE vs. SPRAY (*p* < 0.05). Mean ± SD.

The condition x time interaction was not significant for any skin temperature. Significant (*p* < 0.05) main effects existed for time (T_NECK_: F = 10.77, T_CHEST_: F = 11.16, T_ARM_: 49.32, T_LEG_: F = 7.58) and condition (T_NECK_: F = 4.24, T_CHEST_: 3.41_,_ T_ARM_: 3.45). Pairwise comparisons revealed the following. T_NECK_ following RI1 was significantly lower during ICE than SPRAY (*p* < 0.05) (Figure 2). T_CHEST_ was significantly lower during ICE than SPRAY (*p* < 0.05) and tended to be lower than CON (*p* = 0.08, *d* = 0.48) following RI1 (Figure 2). T_CHEST_ was significantly lower (*p* < 0.05) during ICE than both SPRAY and CON both immediately following HEX2, and after RI2. T_ARM_ tended to be lower during ICE compared to CON and SPRAY, both immediately following HEX2 (*p* = 0.07; *d* = 0.59, *p* = 0.08; *d* = 0.63, respectively) and after RI2 (*p* = 0.08; *d* = 0.44, *p* = 0.08; *d* = 0.80, respectively). T_LEG_ did not change at any time point, for any condition.

### 3.2. Expired Gas and Heart Rate

No significant differences existed among treatments at any time point in metabolic and cardiovascular variables. Thus, data were collapsed between treatments and reported across time (Table 1).

### 3.3. Perceptual Measures

#### 3.3.1. Thermal Sensation

**Figure 3:**
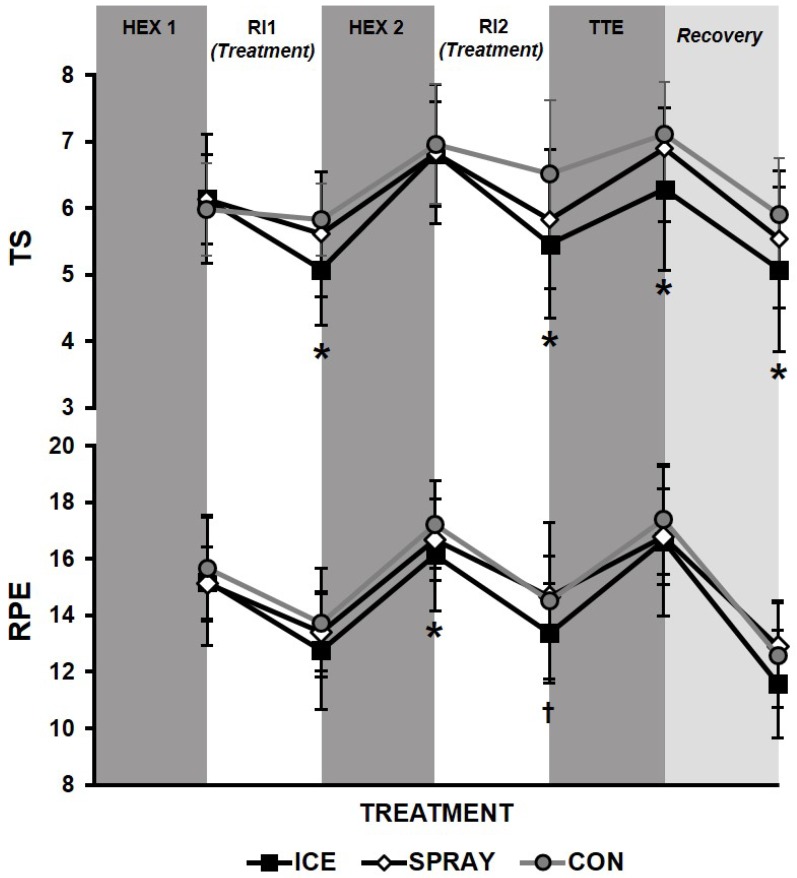
Perceptual responses to repeated exercise bouts (HEX: high-intensity exercise interval; TTE: time-to-exhaustion) with intermittent rest intervals (RI) and neck cooling treatments (ICE: “wet ice” bag; SPRAY: non-thermal cool spray; or CON: control). TS: thermal sensation; RPE: rating of perceived exertion. Significance among treatments: * ICE vs. CON (*p* < 0.05), ^†^ ICE vs. SPRAY (*p* < 0.05). Mean ± SD.

The condition x time interaction was significant (F = 2.717, *p* = 0.034). A significant main effect existed for time (F = 19.46, *p* = 0.001) and condition (F = 4.22, *p* = 0.029). Pairwise analysis showed participants felt significantly cooler during ICE than CON (*p* = 0.02) and tended to feel cooler than during SPRAY (*p* = 0.10). Analysis of the simple main effects showed TS was the same among treatments immediately after HEX1 and HEX2. However, following RI1 and RI2 (i.e., after treatment administration), TS decreased (i.e., participants felt significantly cooler) with ICE compared to CON (*p* < 0.05; Figure 3) and tended to decrease with ICE compared to SPRAY after RI1 (*p* = 0.10; *d* = 0.35). SPRAY tended to have a similar cooling affect as ICE following RI2 when compared to CON (*p* = 0.07, *d* = 0.67). Immediately following TTE, TS significantly (*p* < 0.05) decreased during ICE compared to CON and tended to decrease compared to SPRAY (*p* = 0.09; *d* = 0.58). This continued through recovery as ICE was significantly lower than CON (*p* < 0.05).

#### 3.3.2. Rating of Perceived Exertion (RPE)

The condition x time interaction was not significant (*p* = 0.519). A significant main effect existed for time (F = 44.808, *p* < 0.000) and condition (F = 6.26, *p* = 0.040). Pairwise analysis showed participants felt significantly less exerted during ICE than CON (*p* = 0.018). Analysis of simple main effects showed RPE was the same among treatments immediately after HEX1 (Figure 3). Additionally, no differences existed among treatments after RI1. However, RPE was lower in ICE following HEX2 when compared to CON (*p* < 0.05) and tended to be lower when compared to SPRAY (*p* = 0.10; *d* = 0.38). RPE was also lower with ICE compared to SPRAY and tended to be lower compared to CON (*p* = 0.08; *d* = 0.41) after RI2. RPE was not different between treatments immediately following TTE but tended to be lower in ICE during recovery compared to SPRAY (*p* = 0.10; *d* = 0.40) and CON (*p* = 0.12; *d* = 0.37).

### 3.4. Time To Exhaustion

Treatment did not influence TTE (ICE = 66.5 ± 16.6, SPRAY = 74.0 ± 23.7, CON = 65.9 ± 16.6 s).

## 4. Discussion

Our findings provide new insight into the relationship between neck cooling, physiological and perceived fatigue, skin temperature, and actual performance during repeated, high-intensity exercise intervals. These data show that holding an ice bag on the back of the neck for one minute between rounds of HEX attenuates the rise in skin temperature and, as a result, makes participants feel cooler and, at times, less exerted, but does not alter cardiovascular or metabolic function. Thus, intermittent neck cooling may function as an effective, practical, and legal cooling option for combat sport athletes (or others who participate in repeated HEX for sport or recreation) to utilize during competition and training.

### Temperature, Perception, and Performance

Temperature, perception, and actual exercise performance significantly influence each other [20]. In fact, Mündel [4] suggested the temperature of a skin surface area ≤10% of the body may significantly effect hormonal and perceptual responses to exercise [9]. The attenuated rise in neck, chest, and arm skin temperature seen here with ICE did not correspond with a reduced rise in heart rate (or altered metabolic function) as proposed by others [20]. However, the ability of ICE to cool the skin is similar to previous research [21] and likely explains our improvements in TS [13]. This reduction in TS allowed participants to enter subsequent exercise bouts feeling cooler. Interestingly, while the effect of the first application of ICE on TS was eliminated by the next round of exercise (i.e., post HEX2 TS was not different between treatments), its effects on RPE persisted (i.e., post HEX2 RPE was significantly lower in ICE). This continued through RI2, suggesting that participants both (1) completed the standardized exercise bout (HEX2) and (2) went into the third bout of exercise (TTE) feeling less exerted and cooler, even though several cardiovascular and metabolic markers (RER_PEAK_, VE_AVE_, HR_AVE_, and HR_PEAK_) indicated HEX2 was more physiologically challenging than HEX1. They also felt cooler after a matched workload (TTE) and 2 min into recovery. Additionally, lower pre-exercise TS corresponded with lower post-exercise RPE (when compared to a standardized work bout—HEX2). Unfortunately, our study design eliminated the ability to determine if the reduced post-RI2 RPE was a function of ICE (1) aiding recovery from HEX1; (2) pre-cooling the neck in preparation for HEX2; (3) or both. Research from others suggests intermittent cooling of other body parts reduces RPE [22], but we are the first to show such changes with intermittent neck cooling.

The ability of ICE to attenuate the rise in skin temperature diminished as the exercise session extended. This means changes in skin temperature either (1) did not fully explain the continual decrease in TS or RPE; or, more likely, (2) caused a continued perception of cooling, even when actual cooling was not occurring. Actual temperature changes are not required to alter thermoregulatory responses or to improve performance [7]. We chose to globally account for both actual and perceived cooling by including a treatment group that received an over-the-counter spray (SPRAY) containing menthol, a substance that interacts with cold receptors in sensory neurons [6] and alters exercise performance [5,23]. Recent evidence suggests an L-menthol spray with a concentration of 0.2% lowers TS [23]. Our data show a limited effectiveness of SPRAY, which is probably a result of differences in exercise protocols (i.e., our repeated HEX vs. 40 min of steady-state) [5,23], or our use of an 8% commercially available product. None of our participants reported skin irritation or discomfort from SPRAY, as has been reported in some previous studies. Thus, it was unlikely to explain our findings.

Changes in physical and perceptual markers during intermittent [13,14] or pre-cooling [20] of the actual exercising muscle, face and palm, and/or neck usually correspond with improvements in endurance and time trial performance [24]. The inability of ICE to influence leg temperature and subsequently improve TTE in our study is most likely explained by a combination of (a) the location and (b) a short duration (1 min) of cooling, and the (c) high-intensity and (d) short duration (~1 min) of TTE. Cooling the legs directly would obviously maximize the influence on skin temperature and likely improve TTE performance [14]. However, this strategy is unrealistic for combat sport athletes or during any exercise bout that uses multiple muscle groups. As opposed to previous research, which typically use >5 min [24,25,26] or even leaves the cooling device on the neck during the actual exercise [17], our cooling period was limited to 1 min. This distinction is important as the amount of cooling time significantly alters its effectiveness [27]. Our time was chosen as it reflects the timeframe of combat sports, and excessive cooling may actually harm repeated sprint performance [26].

Cooling is typically effective for exercise tasks performed at 40–70% VO_2_max that last ~20–60 min [14,17], but not always [15]. We are the first to examine intermittent neck cooling at normal room temperature at this high of exercise intensity. Drust et al. (2005) showed that performance during a 40-min high-intensity, intermittent cycling protocol (15 s on, 15 s off) was worse when performed in a hot room compared to a normal temperature room [28]. Moreover, the effects of intermittent cooling during high-intensity exercise may be enhanced when in hot environments. Individual perception of recovery, independent of physiological measures [27], relates to subsequent sprint performance [29]. Therefore, the fact that our reduced TS persisted through HEX2 suggests that RPE and performance might have actually improved if a standardized HEX was performed instead of the short TTE (TTE required maximal effort, eliminating our ability to compare the effects of ICE during RI2 on post-TTE RPE), or had additional rounds of HEX been performed. The combination of short duration, high RPE, and reduced cardiovascular or metabolic demand (which was significantly greater in HEX2, but not TTE, compared to HEX1) suggest that failure during TTE was a muscle, not cardiovascular issue. The apparent delayed improvements in perception caused by reduced skin temperature means intermittent cooling might enhance performance in exercise tasks lasting longer than were tested here.

In concert with previous literature, our data suggest ICE might be effective for combat sports or other activities that require repeated, high-intensity exercise intervals that last more than two rounds and result in high physiological and perceived fatigue ratings [2]. ICE could also help long-term training motivation as how one (athlete or non-athlete) feels during exercise predicts future exercise intentions and behavior [30], which is probably why RPE is regarded as a highly effective tool for monitoring combat sport training [31]. The specific mechanisms explaining our findings are unknown. Several postulates exist (e.g., altered sensory perception, pain reduction, Gate Control Theory, and Central Fatigue Theory) and have been reviewed elsewhere [14].

Not measuring core temperature during our study was a limitation. However, extensive research on humans exercising at normal environmental temperatures supports our position [7,17,18]. Specifically, one study showed neck icing does not significantly alter core temperature more than simple passive rest [32]. Improvements in exercise performance have even been noted with cooling when core temperature did not change [25] or even increased [33]. Moreover, core temperature changes slower than skin temperature, making skin the more likely primary thermal signal [18]. We also acknowledge that our exercise task was atypical for exercise physiology research and would be considered novel to most participants. This left us potentially susceptible to a familiarity effect during visits 3 and 4. With this in mind, we specifically utilized participants with a history of frequent, high-intensity exercise intervals. We also analyzed and confirmed that no depended variables were altered by visit order (*p* > 0.4).

## 5. Conclusions

Small durations (i.e., 2 min total) of intermittent wet-ice neck cooling (i.e., an ice bag) partially attenuated the rise in skin temperature during repeated bouts of high-intensity exercise. This made participants feel cooler and less exerted (at times) over the course of 12–15 min (total) of exercise. However, time to exhaustion during a ~1 min cycling protocol was not altered. The benefits of neck cooling may be limited for activities primarily limited by power or muscular endurance. However, it may benefit combat sport athletes or other individuals during exercise of more than two, 5-min HEX rounds. Practitioners may incorporate intermittent neck cooling as an effective, practical, and legal cooling option for combat sport athletes. Future research should focus on physiological and performance outcomes during actual competition.

## Figures and Tables

**Table 1:** The cardiovascular and metabolic variables collapsed between treatments and displayed across time.

Variable	HEX 1	HEX 2	TTE
RER_AVE_	1.00 ± 0.5	0.97 ± 0.03	1.01 ± 0.04
RER_PEAK_	1.12 ± 0.07	1.22 ± 0.09 *	1.13 ± 0.07
VE_AVE_ (L·min^−1^)	67.6 ± 12.8	80.3 ± 16.1 *	75.1 ± 17.0 ^¥^
VE_PEAK_ (L·min^−1^)	89.8 ± 17.5	90.9 ± 18.1	89.9 ± 19.1
HR_AVE_ (bpm)	147.7 ± 17.7	163.6 ± 14.0*	157.3 ± 15.7
HR_PEAK_ (bpm)	169.7 ± 11.2	175.7± 10.6 ^§^	173.3 ± 11.4
VO_2AVE_ (mL·kg^−1^·min^−1^)	35.5 ± 5.9	39.0 ± 6.3	32.6 ± 6.5
VO_2PEAK_ (mL·kg^−1^·min^−1^)	45.1 ± 6.1	44.8 ± 7.5	41.7 ± 8.2

Data are reported as mean ± SD. * signifies *p* < 0.05 compared to HEX 1 and TTE, ^§^ signifies *p* < 0.05 compared to HEX 1, ^¥^ signifies *p* = 0.07 compared to HEX 2.
